# Photothermal Effectiveness of Magnetite Nanoparticles: Dependence upon Particle Size Probed by Experiment and Simulation

**DOI:** 10.3390/molecules23051234

**Published:** 2018-05-22

**Authors:** Robert J. G. Johnson, Jonathan D. Schultz, Benjamin J. Lear

**Affiliations:** Department of Chemistry, The Pennsylvania State University, University Park, PA 16802, USA; johnrobe94@gmail.com (R.J.G.J.); jonathanschultz2022@u.northwestern.edu (J.D.S.)

**Keywords:** photothermal, nanoparticles, heat transfer

## Abstract

The photothermal effect of nanoparticles has proven efficient for driving diverse physical and chemical processes; however, we know of no study addressing the dependence of efficacy on nanoparticle size. Herein, we report on the photothermal effect of three different sizes (5.5 nm, 10 nm and 15 nm in diameter) of magnetite nanoparticles (MNP) driving the decomposition of poly(propylene carbonate) (PPC). We find that the chemical effectiveness of the photothermal effect is positively correlated with particle volume. Numerical simulations of the photothermal heating of PPC supports this observation, showing that larger particles are able to heat larger volumes of PPC for longer periods of time. The increased heating duration is likely due to increased heat capacity, which is why the volume of the particle functions as a ready guide for the photothermal efficacy.

## 1. Introduction

The photothermal effect of nanoparticles, whereby light is absorbed and converted to thermal energy, has emerged as an attractive option for driving thermally-activated physical and chemical transformations at greatly enhanced rates [1,2,3]. For strongly-absorbing and weakly-emissive nanoparticle systems, it is possible to use widely-available light sources to bring the nanoparticles to extreme temperatures [4]. Because of the nanoscale nature of the photothermal agents, these extreme temperatures are localized in both time and space and are well matched to the scale of the elementary steps of molecular transformations [5]. Additionally, the localized nature of the heating means that while the immediate surroundings of the nanoparticles experience extreme temperatures, the bulk of the system experiences minor temperature fluctuations, limiting the extent of unwanted processes [6]. Nevertheless, the localized heat remains effective at driving chemical transformations, and to date, the photothermal effect of nanoparticles has proven to be extremely effective in various applications [7], including the ablation of cancerous cells without damage to the surrounding tissue [8,9,10], in vivo drug delivery [11], selective defect healing in polymers [12,13], decomposition of molecules, [3,14,15], regeneration of CO${}_{2}$ [16], killing of bacteria [17] and cross-linking of polymer networks [1].

Although the above applications are exciting, it is not well understood how thermal diffusion behaves at the nanoscale [18], nor how the properties of the nanoparticles, such as size, geometry, specific heat and ligand identity, can be used to control the generation and dissipation of thermal energy. This lack of knowledge inhibits efficient design and optimization of photothermal agents, preventing full realization of the promise this approach has for controlling chemical transformations with molecular-scale precision in time and space.

To a large extent, the knowledge gap concerning the influence of nanoparticles’ physical and chemical properties over photothermally-driven reactions is because of the prevalence of gold nanoparticles (AuNPs) in the literature. AuNPs’ popularity arises due to the fact that AuNPs of a certain range in diameter possess a strongly-absorbing plasmon resonance, while being non-emissive. Indeed, these desirable optical properties have led to a large literature involving the photothermal effect of these particles, and the mechanism of photothermal heating for plasmonic particles is now well established [19,20,21]. However, because of the low melting point of gold, which is further exaggerated for nanoscale particles (600 K for 2 nm AuNPs) [22], the propensity for Coulombic explosion in larger particles [23] and relatively weak binding of surfactants (thiols, amines, citrate, etc.) [24,25], the optical, physical and chemical properties of AuNPs are unstable under the photothermal conditions required to drive thermal reactions at extreme rates [4,26,27,28]. This lack of stability of AuNPs prevents systematic studies of the influence of various nanoparticle properties on photothermal efficacy.

**Figure 1:**
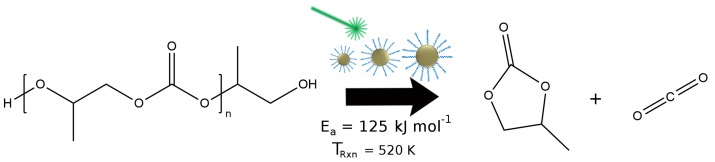
Decomposition reaction for poly(propylene carbonate).

Fortunately, the number of nanoparticles demonstrated to be effective photothermal agents is increasing and includes more thermally-stable examples such as magnetite (Fe${}_{3}$O${}_{4}$) [29], Cu${}_{2-x}$S [30], Cu${}_{2-x}$Se [31], PbS and carbon black [32]. Our lab has previously shown that magnetite nanoparticles (MNP) are able to drive high barrier reactions, such as the clean decomposition of polypropylene carbonate(PPC), while maintaining their overall size, shape, composition, structure and ligand environment [33]. In addition, one can synthesize MNP in multiple sizes [34,35,36], and combined with their stability, this makes MNP an ideal system for investigating how the properties of nanoparticles control nanoscale heating. In this work, we focus on the effect MNP size has upon the decomposition of PPC (Figure 1) and find that, of the properties considered, the extent of the reaction is best correlated with the volume of the particle. Modeling thermal diffusion around the particle’s surface reveals that larger particles heat a larger volume of PPC to reactive temperatures and the medium remains at a high temperature for a longer duration after irradiation. Both of these behaviors are a direct result of the increased volume (and associated heat capacity) of the particles.

## 2. Results and Discussion

### 2.1. Nanoparticle Stability under Photothermal Conditions

**Figure 2:**
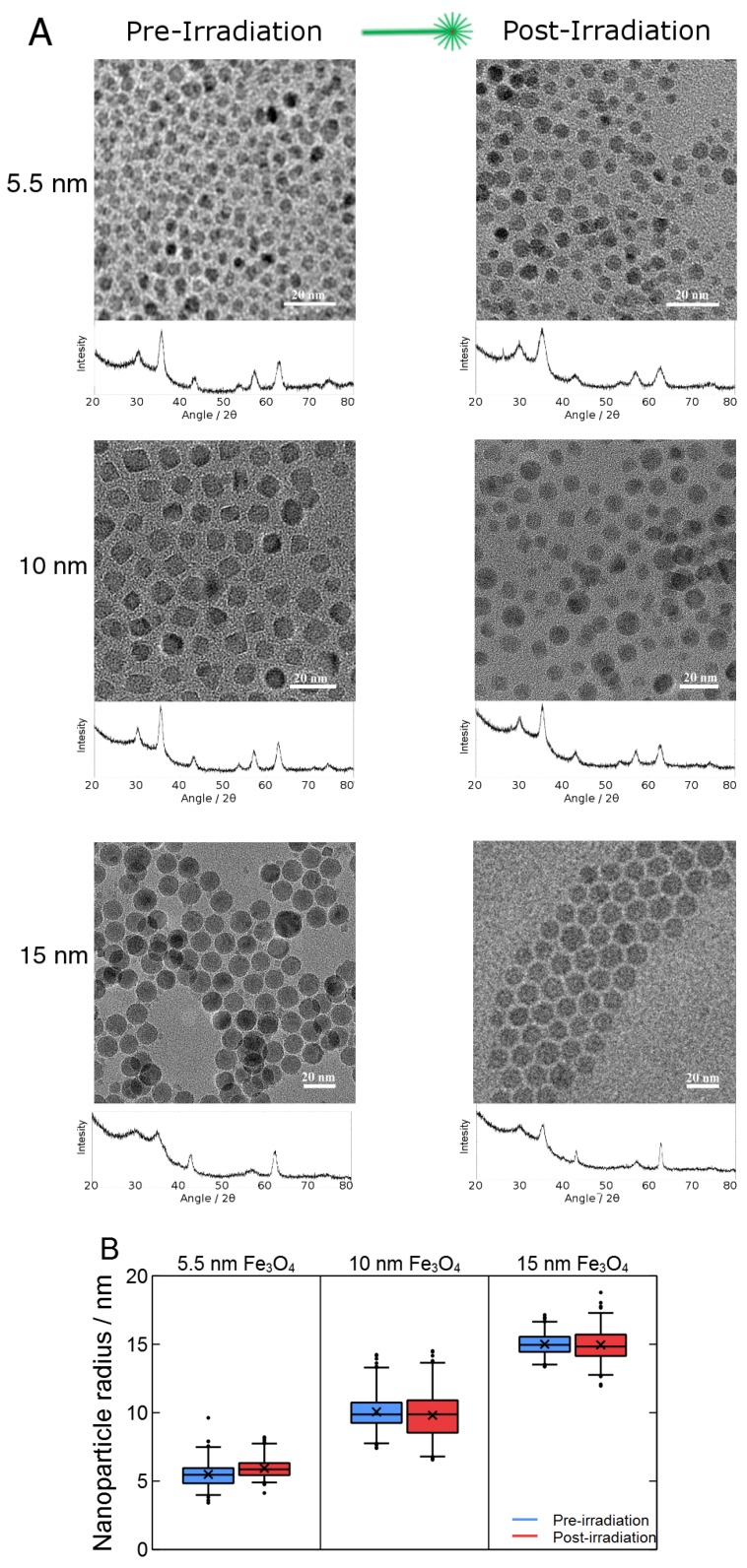
(**A**) TEM images and XRD patterns for magnetite nanoparticles (MNP) synthesized showing geometric and crystalline stability; (**B**) Whisker plots showing various values obtained from TEM images. In these plots, ‘×’ indicates the mean size of the particle, while the horizontal line near ‘×’ indicates the position of the median size. The top and bottoms of the box show the 25th and 75th percentiles of the sizes, while the whiskers indicate the second and 98th percentiles of the sizes. The individual points are the particle sizes that lie outside these bounds.

We synthesized particles of 5.5 nm, 10 nm and 15 nm using the approach outlined in the Materials and Methods Section. To ensure all particles were stable under intense laser irradiation, samples of each particle were characterized by XRD, TEM and IR before and after irradiation by 7000 pulses (8-ns pulse width, 10 Hz) of 532-nm light with an irradiance of 25 MW cm${}^{-2}$. This irradiation was performed on colloidal suspensions in hexanes. As shown in Figure 2, TEM analysis shows that the particles do not experience significant changes in size under these conditions, while the XRD patterns demonstrate that the MNP experience no apparent changes to their crystalline phase. These results are consistent with previous results involving MNP [33] and that indicate that the vast majority of heat generated by the particles is effectively dissipated to the surroundings without driving significant undesirable changes o the particles such as fragmentation, agglomeration or crystalline phase transitions. This stability in terms of size, shape and structure allows us to systematically probe how differences in MNP size affect their ability to photothermally drive the decomposition of PPC, which would not be possible with AuNPs under these photothermal conditions.

### 2.2. Photothermal Decomposition of PPC

**Figure 3:**
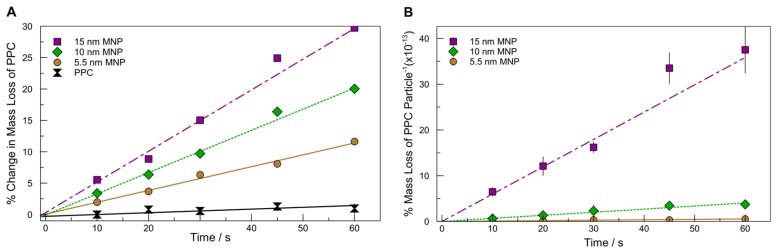
(**A**) Percent mass loss over time for MNP and neat PPC; (**B**) Percent of PPC decomposed for a single particle over time.

In order to determine the relative efficacy of these particles for photothermal decomposition of PPC, we cast PPC films of 0.99% weight MNP. We then exposed these films to the same irradiance used to test the MNP stability and followed the course of the PPC degradation over time, using the mass loss of the composite film as a metric for the course of the reaction [33,37]. Figure 3A plots the course of the reaction for four samples exposed to laser irradiation: pure PPC, PPC:5.5 nm MNP, PPC:10 nm MNP and PPC:15 nm MNP. The mass loss throughout time is linear, so we performed a least squares linear fit to the data. The slope of this fit is proportional to the instantaneous rate of the decomposition and can be used to establish the relative efficacy of the MNP as photothermal agents (Table 1). For the ease of visual comparison of the slopes, the data have been shifted along the y-axis by adding a constant to all points in a series such that the fits pass through the origin. Because we are only concerned with the comparison of slopes and because we are adding a constant to the data, this does not alter our interpretation nor conclusions. The as-collected data are tabulated in the Supplementary Information (Table S1).

From Figure 3A and Table 1, it is obvious that all irradiated samples containing nanoparticles experience enhanced rates of decomposition. In addition, the enhancement of the rate provided by the MNP is clearly size dependent.

Because all irradiated films contained the same mass fraction of nanoparticles, Figure 3A is a direct comparison of the relative rate of decomposition as a function of nanoparticle mass. However, because the particles vary in size, each sample will not necessarily contain the same number of particles. Using the size and density of the MNP to estimate the number of particles present in each film, we adjusted the plot to compare the rate of decomposition on a per particle basis (Figure 3B, summarized in Table 1). Similar to the results on a per mass basis, as the size of the MNP increases, so too does the efficiency for driving the decomposition of PPC. However, on a per particle basis, the dependence of size is much more apparent than when considering the system on a per mass basis (Table 1).

### 2.3. Nanoparticle Properties Controlling PPC Decomposition

**Figure 4:**
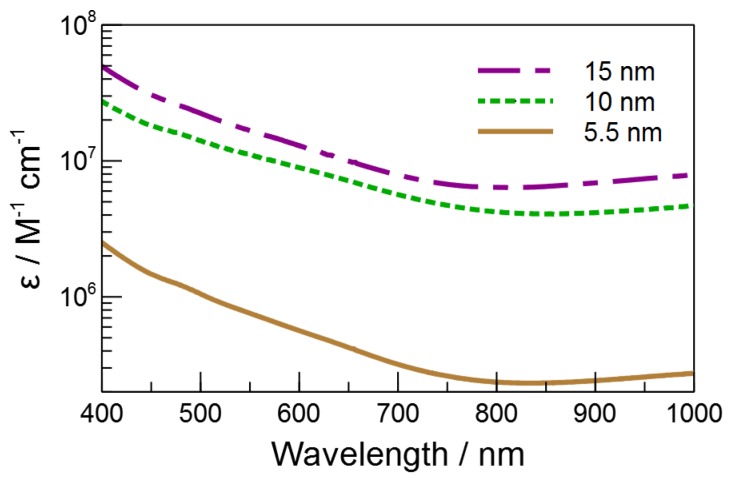
Extinction spectra of the three sizes of MNP.

A number of particle properties are expected to change as a function of size, and several of them could influence the photothermal effect. Perhaps the most obvious parameter to consider is the absorptivity of the particles. As seen in Figure 4 and Table 1, the extinction coefficient at 532 nm of the MNP increases with size. This trend is a result of the fact that we are exciting on the low energy edge of the bandgap transition, and as the particle size increase, the bandgap narrows and the transition red-shifts, increasing the absorptivity [38]. It would follow that if a majority of the light is converted to thermal energy, we would expect the decomposition rate to correlate with the absorption coefficient. At the same time, the effectiveness of the nanoparticle might be expected to depend on the surface area and volume of the particle. As the surface area increases, so too will the rate at which thermal energy is transferred to the surroundings and the efficiency with which the surroundings’ temperature is raised. Finally, as the volume increases, so too will the heat capacity of the particle, which means that the particle will have greater overall capacity for transferring thermal energy to the surroundings.

**Figure 5:**
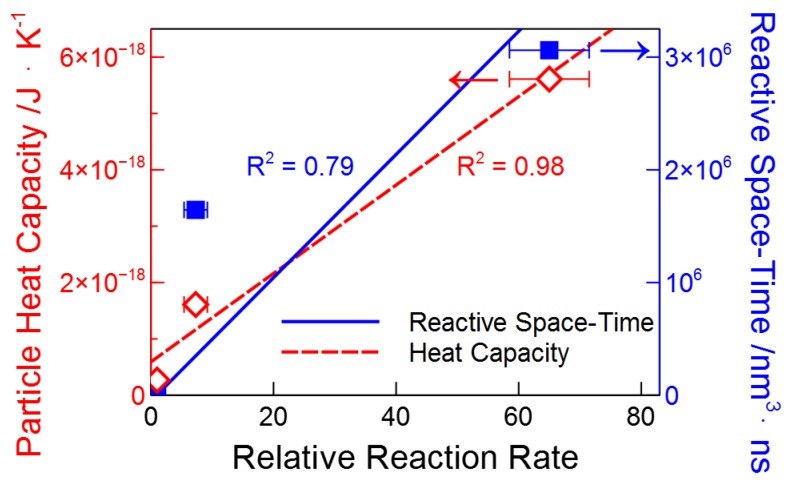
Reaction rate as compared to the heat capacity of a single particle and the reactive space-time, as well as linear least square fits to these parameters.

When looking at the correlations between these properties of the nanoparticles ($\epsilon_{532}$, *r*, $r^{2}$, $r^{3}$) and the relative per particle slope (Table 1), we find that the strongest correlation is with the volume of the particle. This correlation is shown in Figure 5. The examined correlations are found in the Supplementary Information (Figure S2). Although a correlation between photothermal efficacy and volume is rational due to the increase in per particle heat capacity, we performed numerical simulations to help validate the observed correlation.

### 2.4. Simulation of Photothermal Heating

**Figure 6:**
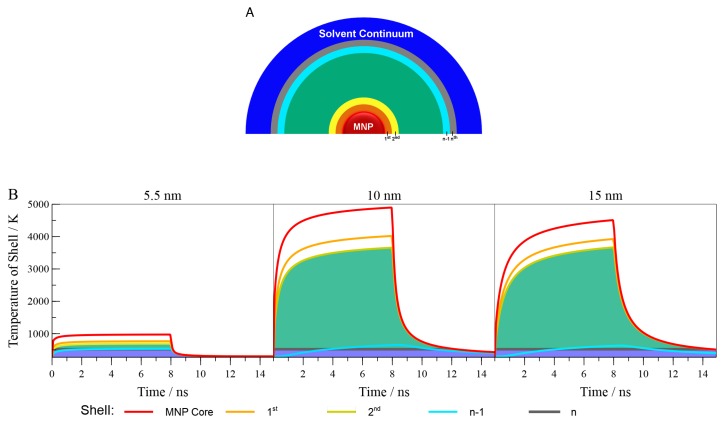
(**A**) Representation of the model used for the simulations where n is the number of shells through which the heat moves; (**B**) Results of the simulation showing the temperature of selected shells for 5.5 nm, 10 nm and 15 nm MNP.

Simulations of heating by the irradiated particles followed the procedure outlined in the Materials and Methods Section. In short, the nanoparticles are treated as a heat source, where the absorption cross-section of the nanoparticles and the irradiance of light are used to calculate the heat flux at the nanoparticles surface, assuming that all light absorbed is converted to thermal energy. This assumption is made to allow the comparison between calculations, though it should be noted that the quantum yield of the non-radiative pathway is not known for these particles. This thermal energy is then allowed to flow through successive shells of the medium (Figure 6A), which in this case is PPC. From this simulation, we obtain the temperature of each discrete shell at all time points through which the simulation runs.

Before discussing the results of these simulations in detail, it is necessary to address the shortcomings of this approach. First and foremost, the equations that the simulations are based on are only valid for macroscopic systems. On the nanoscale, heat transfer is much more complicated and is not understood well enough to allow for accurate simulation [18]. Thus, we use these simulations as a first approximation. Second, we do not include the surfactant layer on the nanoparticles, which would provide a shell with a different thermal diffusion. However, even for the smallest particles, thermal diffusion extends far beyond this surfactant layer, and so, we expect the exclusion to lead only to small errors. Finally, we do not explicitly address thermochemical considerations within the PPC medium itself. The decomposition of PPC is endothermic and, were the reaction explicitly included, should contribute to the apparent dissipation of thermal energy. However, despite these caveats, all simulations we investigated shared these same limitations, and so, we believe they can provide rough quantitative guidance for comparing samples and understanding the influence of MNP properties on photothermal efficacy. This reasoning is supported by the fact that the peak temperatures predicted by these simulations (Figure 6B) are of a similar order of magnitude to those estimated from earlier considerations of the rate of PPC decomposition under photothermal conditions [33].

**Figure 7:**
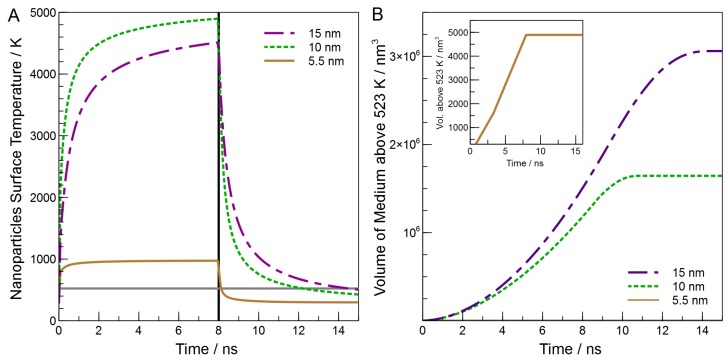
(**A**) Heating and cooling of the nanoparticles’ surface as simulated; (**B**) Reactive volume of PPC as the simulation proceeds. The inset is the volume of the medium for the 5.5 nm MNP.

We begin with the discussion of the simulation results by noting that no matter the size of the particle, the heating and cooling portions of the simulation have a similar shape (Figure 7A). Indeed, while the laser is on, the particles approach a steady-state temperature at which the energy flux out from the particle matches that from absorption of the laser. For the 5.5 nm particles, this steady-state temperature is reached, while for the 10 nm and 15 nm particles, the steady-state temperature is not reached. Interestingly, we find that the simulated maximum temperature is largest for the 10-nm particles, despite the fact that the extinction coefficient for the 15-nm particles is the largest. This is likely due to multiple factors, such as increased surface area for the 15-nm particles, which would lead to quicker dissipation of energy. Because we assume a uniform internal temperature of the particle, only the surface area is important for heat transfer, and not the surface area to volume ratio. In addition, though the extinction coefficient increases with size, so does the heat capacity. These combined effects dictate that the final temperature attained by the particles should be related to the product of $\epsilon_{532}$, $A_{surface}$, and $C_{p}^{-1}$. As can be seen in the Supplementary Information (Figure S3), we do see a strong correlation (*R*${}^{2}>$ 0.99) between this quantity and $T_{max}$.

As might be expected from this discussion, the rates of heating and cooling of the particles also differ with size, with both the rate of heating and the rate of cooling being the slowest for the largest particles. However, we note that at early times all, particles of all three sizes quickly heat to a temperature above the temperature needed for bulk decomposition of PPC (520 K, horizontal black line, Figure 7A), but the larger particles remain above this temperature for much longer after the laser pulse ends. This means that the larger particles provide increased reaction time, likely due to increased heat capacity of the larger nanoparticles; as heat capacity increases, the amount of heat dissipated will rise.

Interestingly, though both the peak temperature and the time spent above 520 K (Table 1) should affect the extent of the reaction, we find that neither provide a reasonable correlation with the relative per-particle rates of mass loss (see the Supplementary Information, Figure S2). Instead, it is the volume of the medium that is heated above the reaction temperature and the time for which this volume is heated that underlie the observed reaction rate. Figure 7B plots the volume above 520 K versus time for our three particles. Here, we see that the total volume that lies above 520 K is greater for the larger particles. Integrating the area under these curves gives the volume of PPC heated above 520 K multiplied by the time that they are heated (we term this the ‘reactive space-time’). The reactive space-time provides acceptable correlation ($R^{2}=0.79$) with the relative reactivity (Figure 5), especially given the drastic simplifications of our model. Thus, when changing the size of nanoparticles without changing the material, the relevant parameter to be considered is the volume of the particle, which controls the reaction time per laser pulse, rather than other parameters such as optical absorptivity or peak temperature. Interestingly, logic seems to suggest that the trend of increased photothermal efficacy with increasing size cannot continue ad infinitum (i.e., bulk heating will not give the same results). It remains for future work to identify the limit of the positive trend with particle size.

## 3. Materials and Methods

### 3.1. Sample Preparation

Iron (III) acetylacetonate (Fe(acac)${}_{3}$) (≥99.9%), oleic acid (≥99%), 1,2 tetradecanediol (>99%), poly(propylene carbonate) (M${}_{n}$ ∼ 50,000) and dichloromethane (≥99%) were purchased from Sigma-Aldrich. Octadecene (>90%) and oleylamine (technical grade) were purchased from TCI America. Dibenzyl ether (>99%) was purchased from Alfa Aesar.

MNP were synthesized through the thermal decomposition of an iron (III) salt in the presence of the stabilizing ligand, oleic acid, under an inert atmosphere. All synthetic steps were performed in a three-neck round bottom flask equipped with a condenser, thermocouple and magnetic stir bar. The 5.5 nm MNP were prepared by the method outlined by Sun and co-workers [35]. Briefly, Fe(acac)${}_{3}$ (2 mmol), 1,2-tetradecanediol (10 mmol), oleic acid (6 mmol) and dibenzyl ether (50 mL) were added to the flask and purged with argon for 30 min. The mixture was brought to 200 ${}^{\circ }$C and maintained for 2 h and then brought to reflux (∼300 ${}^{\circ }$C) and maintained for 1 h. The 10-nm and 15-nm nanoparticles were prepared by a modified LaMergrowth method. [34] For this, an iron(III) oleate precursor was prepared in a 100-mL three-necked flask with 3.3 g (9.3 mmol) iron(III) acetylacetonate and 15 mL (47.3 mmol) oleic acid. The reaction was heated to 320 ${}^{\circ }$C, maintained for 30 min and then quenched. The resulting iron(III) oleate was then diluted with octadecene to be 0.22 M. In a separate flask, 2.5 mL oleic acid and 2.5 g docosane were purged and heated to 350 ${}^{\circ }$C. Once the temperature stabilized, the iron (III) oleate mixture was added at a constant rate of 3 mL/h using a KD Scientific syringe pump. MNP size was controlled by varying the amount of iron precursor added. The reaction was quenched 30 and 100 min after the first drop of iron (III) oleate precursor for 10-nm and 15-nm MNP, respectively. All particles were precipitated and washed with acetone and methanol and collected via a centrifuge. All particles were dispersible in organic solvents.

TEM images were obtained with an FEI Tecnai G2 20 XTWIN electron microscope operating at an accelerating voltage of 200 kV. Size distributions were obtained using ImageJ and contained at least 300 particles per sample. A log-normal function was then fit to the distribution (see the Supplementary Information, Figure S1) to obtain the central size and statistical data shown below.

### 3.2. Casting of PPC Films and Measuring Mass Loss

To measure the extent of decomposition, thin films of PPC were cast on clean, pre-weighed slides (massed using a analytical balance with accuracy of +/− 0.1 mg). Both the polymer and the MNP were separately dissolved in dichloromethane and thoroughly mixed to obtain a solution with an MNP:PPC mass fraction of 1:100, or 0.99% wt MNP. The MNP:PPC solution was then drop-cast onto the slide and allowed to dry over night at 25 ${}^{\circ }$C to minimize the mass loss due to the evaporation of residual solvent driven by photothermal heating and ensuring all the thermal energy was being used to drive the decomposition of the PPC. A typical mass of material deposited was on the order of 10 mg. The slide, now with the composite film, was massed once more and exposed to the second harmonic of an Nd:YAG laser (532 nm) for the specified times. After irradiation, the slides were massed again to obtain the mass loss of the film due to the photothermal effect. For each nanoparticle and mass loading, these experiments were performed in triplicate.

### 3.3. Theoretical Model

To gain insight into the factors controlling the photothermal efficiency, we carried out discrete numerical simulations based on Fourier’s law of thermal conduction. In addition, given the extreme temperature that the particles experience, we also accounted for radiative transfer at the particle’s surface [39]. In the simulation, we treat the nanoparticle as a solid mass, surrounded by a series of 0.5 nm shells of the medium, in turn buried in a continuum of the medium, which is held at 298 K (Figure 6A). At the start of these simulations, the entire system is at room temperature, and the laser heating is simulated by adding a heat flux at the MNP surface. The magnitude of this heat flux is determined by the optical and physical properties of the MNP. Using the acquired UV-visible spectra, we calculate the extinction coefficient on a per-particle basis. We then assume that the only significant means of extinction is absorption and that all of the absorbed energy is converted to thermal energy instantaneously. The thermal energy raises the temperature of the particle, with the ΔT calculated using the heat capacity of the particle.

As the particle’s temperature rises, thermal energy is transferred to the surrounding shells. The thermal conductivity of the medium and the size of the shells dictate that our time step be 150 ps [39]. As the simulation is iterated through each discrete time-point, we determine the amount of thermal flux from the *i*-th to the *i*-th + 1 shell ($q_{i\to i+1}$) according to the following equation:

(1) $$q_{i\to i+1}=\left(T_{i+1}-T_{i}\right)k_{medium}4\pi r^{2}\frac{\tau }{l}$$

In this equation, $T_{i}$ and $T_{i+1}$ are the temperatures of the *i*-th to the *i*-th + 1 shells. $k_{medium}$ is the thermal conductivity of the medium (in this case PPC, with $k_{PPC}$ = 0.17117 · W· m${}^{-1}\cdot$ K${}^{-1}$). τ and *l* are the time step size and shell thickness, respectively. For the layer that is the nanoparticle core, we also add in the radiative flux ($q_{rad}$):

(2) $$q_{rad}=\left(T_{np}-T_{surrounding}\right)\left(5.6703\times 10^{-8}\right)\epsilon_{np}4\pi r_{np}^{2}\tau$$

where $\epsilon_{np}$ is the emissivity of the nanoparticle and is taken to be 0.74. Using Equations (1) and (2), we calculate the heat flux for the core and every shell, which provides both the heat transferred in, as well as the heat transferred out. The final temperature of each shell at the end of the time-point is then arrived at by accounting for the heat capacity of the shell and the net heat flux.

This simulation is iterated for the desired time of the experiment. During the simulation, the heat flux at the nanoparticle can be turned on or off, in order to represent the start and end of the laser pulse. Results for simulations of 5.5 nm, 10 nm and 15 nm MNP in PPC, exposed to a single 8 ns pulse of 532 light (25 MW/cm${}^{2}$) are shown in Figure 6B. In these plots, the laser pulse begins at *t* = 0. The simulations then proceed through the 8 ns of the laser pulse and for another 7 ns to allow complete cooling of the system for the larger particles to the original temperature.

## 4. Conclusions

We have shown that the rate of photothermal decomposition of poly(propylene carbonate) is dependent on the size of the magnetite nanoparticle photothermal agents. In doing so, we highlight that when choosing a photothermal agent, it is important to take into consideration physical properties of the particle in addition to its optical properties. It was shown that reaction rate is most directly related to the volume (heat capacity) of the nanoparticles, rather than the absorptivity, surface area or even temperature attained by the individual nanoparticles. The dependence on the volume is attributed to the fact that particles with larger heat capacities heat larger volumes of their surrounding media for longer periods of time, as supported by the simulations of photothermal heating.

## Figures and Tables

**Table 1:** Collection of various experimental and theoretical values discussed herein. In this table, $\epsilon_{532}$ is the extinction coefficient at 532 nm, C${}_{p}$ is the heat capacity of a single particle, *T*${}_{max}$ is the maximum temperature the particle reached during simulated photothermal heating and *t*${}_{T_{520}}$ is the time the particle was above the reactive temperature for the decomposition of PPC, poly(propylene carbonate).

2*Condition	Relative Slope	Relative Slope	$\epsilon_{532}$	C${}_{p}$	*T*${}_{\max}$	$t_{T_{520}}$	Reactive Space-Time ${}^{‡}$
	(per unit mass) *	(per particle)	M${}^{-1}\cdot$cm${}^{-1}$	J/particle·K	K	ns	nm${}^{3}\cdot$ ns
Pure PPC	0.08 ± 0.063	–	–	–	–	–	–
5.5 nm MNP	1.0 ± 0.04	1.0 ± 0.10	8.43 × 10${}^{5}$	2.69 × 10${}^{-19}$	970	8.1	4.89 × $10^{3}$
10 nm MNP	1.8 ± 0.07	7.3 ± 1.9	1.20 × 10${}^{7}$	1.61 × 10${}^{-18}$	4950	10.9	1.64 × $10^{6}$
15 nm MNP	2.6 ± 0.11	65 ± 6.5	1.84 × 10${}^{7}$	5.61 × 10${}^{-18}$	4600	14.4	3.06 × $10^{6}$

* These are the slopes directly obtained from Figure 3A. ${}^{‡}$ This quantity is used to describe the total volume heated multiplied by the time heated.

## Supplementary Materials

The following are available online: Table S1: As collected mass losses of PPC. Figure S1: Histograms of particle sizes, Figure S2: Correlations between relative photothermal efficacy and peak particle temperature, absorption coefficient and time spent above 520 K, Figure S3: Correlation between max particle temperature and $\epsilon \cdot A\cdot C_{p}^{-1}$, Table S1: Mass losses observed.

## Abbreviations

The following abbreviations are used in this manuscript:

MNP	magnetite nanoparticles
PPC	poly(propylene carbonate)
