# Supplementary material for: Photothermal Effectiveness of Magnetite Nanoparticles: Dependence upon Particle Size Probed by Experiment and Simulation
Source: Molecules. 2018 May 22;23(5):1234. doi: 10.3390/molecules23051234 (PMC6100115; doi:10.3390/molecules23051234)
Supplement: Supplementary file 1 [file molecules-23-01234-s001.pdf]

Supporting information for: Photothermal Effectiveness of Magnetite Nanoparticles: Dependence upon Particle Size Probed by Experiment and Simulation

Robert J.G. Johnson, Jonathan D. Schultz, and Benjamin J. Lear*

Department of Chemistry, The Pennsylvania State University, University Park

E-mail: bul14@psu.edu

Using the Simulations

Simulations used within this text were written in Python. The simulations refer to an input.csv file from which is read various inputs such as the length of the simulation, the size of the discrete shells, initial temperature, wavelength and power of irradiance, etc. The input file must be in the same folder as the script. It is important to note that specific inputs are preprogrammed for certain particles and mediums. Currently, magnetite (all three sizes) and gold (2 nm) are accounted for in the script. As for mediums available, water, hexanes and PPC are written in the script. For the sizes of the particles discussed, user inputs for their properties will be overridden with experimental data. However, the simulation will use the material properties to carry out the simulations if other sizes are desired. For particles not accounted for in the program, absorption efficiency is calculated using Mie theory.

When the simulation is run, the system starts at the temperature it reads from the input file. It then steps through time and calculates the energy absorbed by the particle during the

*To whom correspondence should be addressed

time increment, and based on the extinction coefficient specific heat of the particle, a new temperature for the particle is calculated. The energy is that dissipated based on radiative and conductive heat transport to the surrounding medium for the first discrete shell. This is done by averaging the temperature of the start of this shell and the end of this shell. This is then repeated for the next discrete shell. This is repeated until the n shell is reached, which is described as not having a significant temperature difference as the n-1shell, indicating that the energy the particle has absorbed does not dissipate that far. This is written to an array, and this repeated for the next time point. Once the simulation has reached the final time point, .csv files are generated, which contain arrays of temperature vs. time, temperature vs. space, and a summary file, which contains the user inputs specified. Currently, the script will also generate similar files for concentration vs. time and space and reaction rate vs. time and space; however, as mentioned in the main text, we have not fully incorporated thermochemical considerations, and these files are not to be trusted.

Conversion between per mass and per particle numbers:

In the main text, we present mass loss in terms of per unit mass, as well as per nanoparticle. The samples we investigated were prepared in terms of mass of nanoparticles:mass of PPC. Thus, mass loss per unit mass of nanoparticles is directly obtained from the sample preparation.

In order to estimate the mass loss per particle, we converted the mass of nanoparticles to the number of nanoparticles, using the density of bulk magnetite and the measured size of the particles.

# of nanoparticles = (mass of nanoparticles)$\times$(volume of a single nanoparticle)$^{-1}\times$(density of a nanoparticle)$^{-1}$

This conversion relied upon the following assumptions:

1. *The particles are spherical in shape, and we can use the measured diameter to calculate the volume of the nanoparticles.* In reality, the particles are not perfect spheres, and so, the true volume of the magnetite present is different from what we calculate.

2. *The density of the magnetite in the particles is the same as that for bulk magnetite.* It could be that, due to high surface energy and the surfactant-nanoparticle interface, the density of the nanoparticles differs from that of the bulk.

3. *We ignore the mass of the surfactant.* This certainly leads to errors, but as we do not know the surfactant coverage, we would need to guess at the mass of surfactant present in the sample. Since this would be a guess, we choose to guess the mass as zero.

Though these are approximations, we believe that using them does yield a reasonable estimate of the number of nanoparticles, working from the mass of the nanoparticles used. At least, we believe, the accuracy is sufficient to allow the interpretation outlined in the paper.

Table S1: Unaltered % mass loss of PPC during irradiation.

Time/s	5.5-nm MNP	10-nm MNP	15 nm-MNP
10	5.34 ± 1.02	6.33 ± 1.16	2.29 ± 0.98
20	7.06 ± 1.49	9.31 ± 2.75	5.61 ± 1.31
30	9.71 ±2.36	12.66 ±4.25	11.8 ±1.40
45	11.45± 2.31	19.32 ±4.34	21.7 ± 2.02
60	15.02 ± 1.11	22.98 ±1.46	26.5 ±1.87

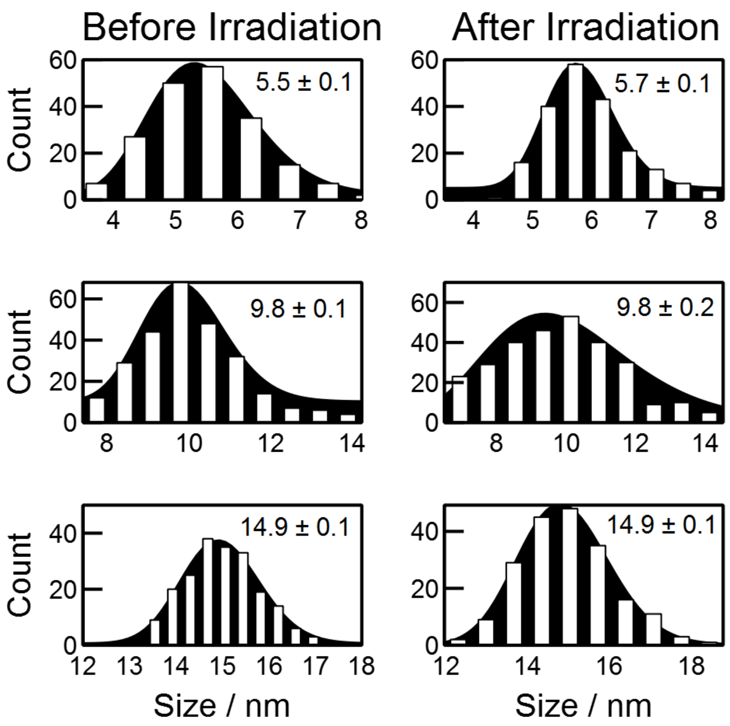

Figure S1: Size distributions and log-normal fits used to statistically obtain values referenced in the text.

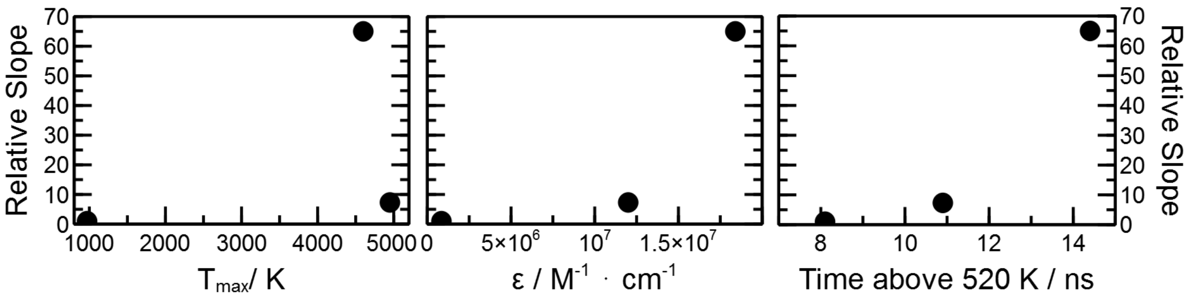

Figure S2: Correlations between variables shown in Table 1 in the manuscript. Left to right are the 5.5-nm, 10-nm and 15-nm MNPs. As is evident, none of these variables give a strong correlation.

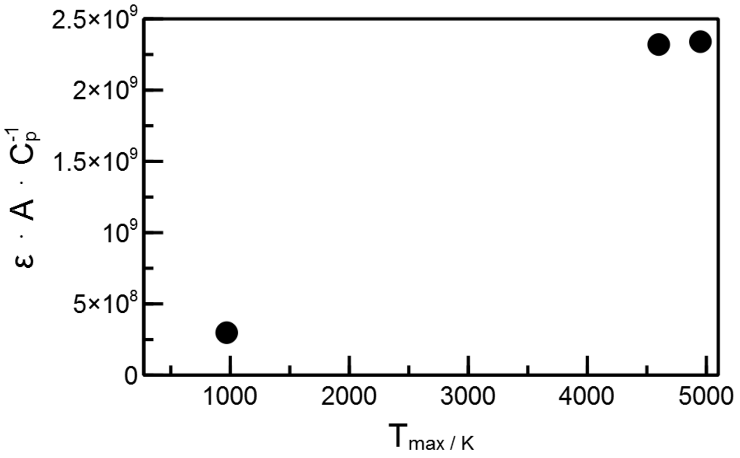

Figure S3: Correlation between the maximum temperature the particles reach and the quantity $\epsilon_{532} \cdot \mathrm{A} \cdot C_p^{-1}$. This represents the particles' ability to absorb and store energy and the surface area that the heat has to diffuse away from the particle.
